# VITAMIN K ANTAGONISM AND CHONDROCALCINOSIS IN THE OSTEOARTHRITIS INITIATIVE

**DOI:** 10.1093/geroni/igz038.217

**Published:** 2019-11-08

**Authors:** M Kyla Shea, Richard F Loeser, Timothy E McAlindon, Sarah L Booth

**Affiliations:** 1 Tufts University USDA Human Nutrition Research Center on Aging, Boston, Massachusetts, United States; 2 University of North Carolina Chapel Hill, Chapel Hill, North Carolina, United States; 3 Tufts Medical Center, Boston, Massachusetts, United States

## Abstract

Calcification of articular cartilage, known as chondrocalcinosis, becomes more prevalent with age. Although chondrocalcinosis can be characteristic of osteoarthritis, it has been associated with more joint pain and disability independent of osteoarthritis severity. Identifying novel modifiable risk factors for chondrocalcinosis may help reduce joint pain and disability. One potential risk factor involves vitamin K because vitamin K-dependent proteins that inhibit calcification are present in articular cartilage. To test the hypothesis that vitamin K antagonism is associated with more chondrocalcinosis, we evaluated the cross-sectional association between warfarin use and chondrocalcinosis prevalence in the Osteoarthritis Initiative (n=1472, 60% female, mean age 64 years). Warfarin is a vitamin K antagonist that interferes with vitamin K-dependent protein function. In a secondary analysis we evaluated whether the prevalence of chondrocalcinosis differed according to dietary vitamin K intake. Chondrocalcinosis of the knee, evaluated using plain x-rays, was detected in 8% of participants. It was three times more prevalent in warfarin users (n=20) compared to non-users [prevalence ratio (95% confidence interval) (PR(95%CI)): 3.02(1.42-6.46); p=0.004, adjusted for age, sex, race, BMI]. Chondrocalcinosis prevalence did not differ according to vitamin K intake [PR(95%CI), compared to tertile 3 (≥189 mcg/d): tertile 1 (<96 mcg/d) =1.30(0.82-2.07), tertile 2 (96-189 mcg/d) =1.17(0.76-1.81), p-trend=0.623, adjusted for age, sex, race, BMI, energy intake]. That chondrocalcinosis prevalence differed according to warfarin use, but not vitamin K intake, suggests vitamin K-dependent protein function may be involved in chondrocalcinosis development. Since warfarin was not commonly used in this cohort, additional studies are needed to substantiate our findings.

